# Occupational therapist’s involvement in social prescribing: A qualitative interview study

**DOI:** 10.1177/03080226241270520

**Published:** 2024-08-19

**Authors:** Gemma Bradley, Beth Atkin, Helen Atkin, Jason Scott

**Affiliations:** Department of Social Work, Education and Community Wellbeing, Northumbria University, Newcastle-Upon-Tyne, UK

**Keywords:** Occupational therapy, social prescribing, qualitative research

## Abstract

**Introduction:**

Social prescribing is a process of helping people to access non-medical activities to promote well-being. For occupational therapists, this is not new, although the social prescribing agenda is creating new roles around these approaches. This study aimed to explore how occupational therapists were involved in social prescribing in the United Kingdom and how they would like to contribute to future developments.

**Method:**

Semistructured interviews were carried out with 19 occupational therapists who identified they were involved in social prescribing activities.

**Findings:**

Thematic analysis led to two over-arching themes: (1) position and identity; and (2) making it work.

**Conclusion:**

Participants perceived similarity with social prescribing, leading to difficulty in positioning occupational therapy alongside this role, emotional responses and identity challenge. Points of distinction between the roles were articulated, including occupational therapy being more medical, having oversight of more complex needs and having more senior roles within teams. To manage workflow, occupational therapists delegate to social prescribing workers, although there is a lack of clarity about competence and varying involvement in supervision. Part of desired future involvement included clearer workflow, occupational therapy involvement in supervision and service development and creating legitimacy for both roles to address social determinants of health.

## Introduction

Social prescribing is a means by which trusted individuals in both clinical and community settings connect people with non-clinical activities to improve health and well-being outcomes (Muhl et al., 2023). In the United Kingdom (UK), the General Practice Forward View (NHS England, 2016) has created opportunities for increased funding and new social prescribing roles, and the agenda was further emphasised within the National Health Service (NHS) Long-Term Plan (NHS England, 2019) which is committed to building the infrastructure for social prescribing in primary care. And while the term ‘social prescribing’ has been popularised in the United Kingdom and gained momentum through explicit policy and funding, action to address healthy ageing, the burden of chronic disease and social determinants of health are global areas concern (World Health Organisation [WHO], 2010, 2023).

Enabling social participation is at the core of the occupational therapy profession and professional bodies, such as The Royal College of Occupational Therapists (RCOT) in the UK, support the key place the profession should have in leading and developing social prescribing services (Royal College of Occupational Therapists [RCOT], 2020). However, it is difficult to understand the extent to which occupational therapy is involved in this agenda; a picture which is made more complex by different terminology and models of service provision. There are calls for occupational therapists to articulate and strengthen their role within this agenda to benefit from the growing momentum and opportunities (Bradley and Scott, 2023).

## Literature review

There is evidence of social prescribing developments in at least 24 countries (Global Social Prescribing Alliance, 2023). Health benefits from social prescribing include improvements in mood and psychological well-being (Chatterjee et al., 2018; Cooper et al., 2022), reduction in social isolation and loneliness (Dayson and Bennett, 2016) and increased resilience (Moffat et al., 2017). Social prescribing can also reduce pressure on General Practice and other health services (Polley et al., 2017), and is receiving greater attention in non-primary care, for example in pre-hospital urgent and emergency care (Scott et al., 2021). Factors affecting adherence and engagement with social prescribing have also been investigated, with accessibility of activities, support with early attendance and leadership with skilled facilitators all highlighted (Husk et al., 2020).

Occupational therapists are one of 14 professions under the umbrella of Allied Health Professions (AHP), and The Royal Society of Public Health (RSPH, 2019) document ‘Driving forward social prescribing: a framework for allied health professionals’ identifies 4 main ways in which AHP can engage with social prescribing. These are active signposting; referring to link workers; being a social prescriber and promoting, growing and developing social prescribing. From a survey of AHP, RSPH (2019) found that 91% of respondents identified signposting as an important part of their job role, over 50% said they did not know how to refer to link workers and only 25% felt they had clear referral criteria for link workers.

Despite examples of social prescribing initiatives which describe engagement in arts, environmental, cultural and physical activities (Husk et al., 2020), evidence-based examples of occupational therapy involvement in social prescribing programmes are lacking. The aims of this study were to explore how UK-based occupational therapists are currently contributing to the social prescribing agenda and how they perceive they could contribute to future developments.

## Method

A qualitative research design was employed using semi-structured interviews to understand experiences of occupational therapists and their involvement in social prescribing. The study has been reported following Consolidated criteria for reporting qualitative research (﻿COREQ; Tong et al., 2007). The core research team consisted of three qualified occupational therapists (female) and one member with a health psychology and public health background (male).

### Recruitment of participants

Information was circulated through RCOT specialist sections, the RCOT primary care network and through UK-based social prescribing networks. Each network had individualised methods of disseminating information, for example through email distribution, e-newsletters and social media. Where appropriate, snowball sampling was used where individuals and networks would re-share information through their own linked contacts.

Occupational therapists were invited to take part if they met the following inclusion criteria: qualified occupational therapist; currently working in one of the four home nations in the UK; self-identified as being in a current role which involved social prescribing. Thirty-nine occupational therapists responded and were sent participant information, written with input from a person with lived experience of health and care services. Following discussion with the research team, two participants felt they were ineligible as their current role did not link to social prescribing. Eighteen participants either did not respond after initial information was sent, or responded after the interview period was complete. Written consent was obtained from all participants recruited to the study.

### Data collection

An interview schedule (See Supplemental Material) was developed by all authors and with input from both a person with lived experience of health and care services and an occupational therapist with experience of working alongside social prescribing link workers.

All interviews were conducted online, using MS TEAMS, by one member of the research team, an occupational therapist with previous experience of conducting qualitative interviewing. The interviewer had no prior relationship with any of the participants, and only the researcher and the participant were present during interviews. Interviews lasted between 28 and 100 minutes, with a mean duration of 48 minutes. All interviews were video and audio-recorded. Video functionality was used to support relationship building during the interview but was not utilised for the purpose of analysis. Audio-recordings were transcribed verbatim with all identifiable information omitted from the transcript. No field-notes from interviews contributed to the analysis.

The interview schedule was not formally piloted, and instead members of the research team met after the first two interviews to review the content of interviews and to consider issues such as the order and wording of questions. Only minor changes to the wording of questions were made at this stage, and all data were included in the final analysis.

### Data analysis

Data were analysed by all authors using reflexive thematic analysis (Braun and Clarke, 2020). This approach to analysis was chosen as it helped to analyse the meaning from each individual participant and then to draw meanings, connections and contrasts across cases. Two analysts carried out early stages including familiarisation with the data and generation of initial descriptive codes. All authors were involved in subsequent stages, with themes generated through a process of interrogating the codes for meaning and meaningfulness and collapsing multiple codes in to overarching themes. Analysis meetings were used to reflect on the coherence of themes, the examples of data which supported themes and the extent to which themes helped to respond to the research aims. The final stages involved defining and naming themes and ordering themes for the purpose of reporting.

Data analysis began while interviews were still ongoing, with one researcher (BA) involved in both activities simultaneously. Concurrent interviews and data analysis assisted the team to recognise similarity in interview responses, suggesting data adequacy (Braun and Clarke, 2021). Analytical software was not used during data analysis with the research team instead using manual methods. Due to time and resource constraints, there was no opportunity to go back to research participants to verify transcripts or to share themes.

### Ethical considerations

Ethical approval was obtained from Northumbria University Research Ethics Committee (ref 45990).

## Findings

Nineteen occupational therapists were interviewed for the study. From the 19 participants, the majority (*n* = 12) were employed as Primary Care Occupational Therapists and are identified as participant numbers 1–12 in the analysis. The remainder of participants were employed under a range of different and often unique, job titles. To protect anonymity, these job titles have not been individually identified, although they are numbered as participants 13–19.

Most participants were employed by the NHS; other employers included a Higher Education Institution, voluntary sector organisations, social enterprises and a community initiative company. Most participants were based in England, with one based in Wales.

From those employed in primary care, 10 were employed at NHS Band 7 (an advanced practitioner level in the NHS, with significant post-qualification experience), one employed at Band 6 (an enhanced practitioner level, with specialist experience often in a particular area of practice) and one employed at Band 5 (a newly qualified practitioner level). Other participant roles were not graded using the NHS banding structure. Participants had a range of post-qualification experience from 2 to 24 years. Participants identified older people, people with long-term health conditions and frailty as the populations they most worked with, although some were more specific, such as working with people with a body mass index over 30 or working with people who were known to be frequent users of services.

Thematic analysis led to two over-arching themes: (1) *Position and Identity* and (2) *Making it work.* Within each theme, there are a number of subthemes. We represent the themes, subthemes and overall narrative in Table 1

**Table 1.** Overview of themes, sub-themes and narrative of themes.

Theme	Subtheme	Narrative
Position and identity	The struggle to position occupational therapy and social prescribing alongside each other	When I put them together – which is one, which is the other? This new thing that focuses on what matters most – but we’ve been doing that for years. They look similar but we cost a lot more. They get to do the bits we’ve never been allowed to. How can I separate out how connected people are to social resources from the rest of my role? All social prescribing has elements of OT, but not all OT is social prescribing.
	The emotional response to identity challenge	I’m screaming ‘that’s occupational therapy’ There is a familiar feeling here – is it because we have always struggled with our own identity? How has the link worker position been allowed to happen?
	Creating distinct positions	The distinct position for social prescribing is knowledge of community activities. Occupational therapy is more medical and more clinical than social prescribing. Occupational therapy should have oversight over patients with complex needs. Occupational therapists have a deeper understanding of meaning of occupation and barriers to participation. Occupational therapists have a more senior role within teams.
Making it work	Managing workflow	Occupational therapists delegate to social prescribers. Delegation is seen as a step down in intensity, after complex issues have been evaluated. Joint and co-working can be needed – it is not necessarily one role, or the other. Similar roles can mean a risk of duplication, confusion and unclear scope of practice. Supervision and caseload oversight is key to managing risk – but there are organisational barriers.
	Imagining a positive future	Positive experience of co-working and joint cases. Occupational therapy can be advocates for meaningful activity and social prescribing. We can lead and manage services and community-level interventions. A call for collective responsibility to promote occupational therapy, starting with professional bodies.

## Position and identity

it’s a means of enabling health . . . of connecting service users to non-clinical activities whether it’s sort of based in community centres or voluntary organisations to . . . and it has the aim of helping people to promote their own health and wellbeing and be better connected to their communities. (Participant 17)

To begin this theme, we use the extract above to note that participants, in the main, were able to articulate definitions of social prescribing clearly. However, when participants attempted to then move on to how this related to occupational therapy, it was here that a clear struggle emerged. This struggle and the subsequent responses are explored in the subthemes below.

### The struggle to position occupational therapy and social prescribing alongside each other

The first extract below provides insight into the struggle which emerges when trying to position social prescribing alongside the profession of occupational therapy:

the minute you ask me about OT and social prescribing together, I kind of feel like my brain goes to sort of jelly with it all, which is one and which is the other, because yeah, it’s really wobbly and on different days I could feel different things. (Participant 9)

Reflection of ‘which is one and which is the other’ is indicative of overlap and similarity that participants found difficult. When articulating the nature of this similarity, some participants linked this to both roles starting from, and subsequently focusing on, what matters most to people. In the extract below, the use of the term ‘bug bear’ in relation to this similarity indicates a challenge that core elements of occupational therapy philosophy are now also fundamental elements of the ‘new thing’ that is social prescribing:

For me this new thing that’s social prescribing, it’s . . . I wouldn’t say it’s a bug bear, that’s probably a bit too strong a word, but I think it is something that really aligns itself well to OT because basically OTs have been doing it for years, like forever and it’s really a core part of our practice those sort of ‘what’s important to you’ conversations that is pretty much the model for social prescribing now. (Participant 1)

This overlapping philosophy, starting from what matters most to people, is also articulated as problematic by Participant 9, who suggests this may then lead to questions about the value of, and requirement for, both roles:

You’ll literally have a social prescriber sat there saying, oh we deal in what matters to the person and you’ve got the OT going, we deal in what matters to you. Well what’s the difference between you? We pay a huge amount for you and not so much for you, we’ll have more of you, thanks. (Participant 9)

Struggle is also reflected within the next extract with the suggestion that valued parts of occupational therapy that the profession have found it difficult to enact in practice are now being undertaken by social prescribing workers:

For me social prescribing is those bits of OT that we’ve always wanted to get involved with, but never been allowed to get involved with because of the roles that we’ve had. (Participant 10)

Another participant suggested that because the roles were similar, they found it difficult to separate activities, and this potentially represented an additional area of struggle:

The social prescribing is sort of a fundamental part of my role in terms of I will also . . . I will always check, you know, how connected patients are, how supported patients are. What are their social resources that are available to them. What social behaviours would they like to have and participate in, that they’re not having access to . . . you can’t kind of separate it off. (Participant 6)

Some participants conceptualised that social prescribing could be thought of as one part of the broader role of occupational therapy, and not seeing this as a ‘bad thing’ suggested not all participants found the position of social prescribing a struggle:

I know OTs don’t have to be involved in all social prescribing, but I think all social prescribing has elements of OT. (Participant 13)

I think occupational therapists need to realise actually social prescribing is just one teeny tiny bit of our role . . . I don’t see that being a bad thing. (Participant 14)

### The emotional response to identity challenge

The narrative which began in the first theme – about how the emergence of social prescribing has led to challenging questions about how this new role is positioned in relation to occupational therapy – is developed in the second theme, which outlines strong emotional responses and a challenge to identity.

I’ve been asked about whether I feel sort of offended that social prescribers have taken part of the OT role. (Participant 6)

When I heard that social prescribers were getting involved in the learning disability care plans, my head was screaming, that’s occupational therapy, that’s occupational therapy. (Participant 10)

Words such as ‘offended’ and ‘screaming’ suggest a powerful emotional response, echoed by other participants who discuss ideas being ‘poached’ from occupational therapy or reflect that some may feel ‘anger’. Another participant responded that social prescribing has ‘lovers and haters’ amongst the occupational therapy profession. The suggestion that social prescribing may be taking part of the occupational therapy role links the emotional response to a challenge to professional identity and is further developed in the extract below:

Are they, you know, being OTs without the name and the training of OTs . . . like stepping on our toes . . . OT has always struggled a bit with identity and recognition and you know, what we do is so holistic and can be so broad that it’s poorly understood. (Participant 17)

One participant expressed a reflection that the RCOT had some responsibility for protecting the profession from a perceived threat from social prescribing roles:

Some of the research that I’ve done, you know, from like the RCOT’s perspective as well is, you know, how is the link worker position been allowed to happen, because obviously a chunk of what OTs do, it’s connecting people and it’s supporting people to engage in what they want to, need to and must do occupations. (Participant 12)

### Creating distinct positions

Building on the first two subthemes, and potentially as a response to struggles with position and identity, occupational therapists found different ways to emphasise points of distinction between their own role and that of social prescribing. From a positive perspective, many participants emphasised the extensive knowledge that social prescribers held about community resources, viewing this as their specialist contribution, and as a point of difference to occupational therapy:

I would say social prescribers have got a better knowledge (of community activities) because, obviously, that’s what they do, that’s their, that’s their sort of speciality. (Participant 3)

Other examples emphasised occupational therapy as a more ‘medical’ and ‘clinical’ role than social prescribing:

(The difference is) the medical issues and I think it’s looking at, yeah, like the long term medical issues, the physical issues and all those comorbidities. . . . It’s having that understanding of those conditions and physical limitations . . . doing those clinical assessments. (Participant 11)

I don’t think they should be doing any of the clinical work and I also do have a little bit of an issue with, they do try and do a lot of prescribing equipment. (Participant 3)

Another point of distinction related to level of complexity, with some participants suggesting that people who were deemed to be complex should be under the oversight of the qualified occupational therapist:

OT tends to see if have multiple complexities, struggling with day-to-day activities, have mobility problems, have had falls. (Participant 1)

So it’s really clear isn’t it, with people with . . . At the top of the Universal Personalised Care Triangle, with the 5% or whatever it is with the most complex thing, really clear that actually they probably need an occupational therapist in order to do some of the things they need, want and have to do in a day. (Participant 9)

Participants made links to theoretical knowledge and reasoning skills as a way of further emphasising points of difference. The first extract below highlights how fundamental concepts of occupational therapy would not be understood in the same way by social prescribers and refers to less complex reasoning process used by social prescribers. The second extract develops this and reflects how theoretical underpinning enables occupational therapists to analyse occupations in a different way:

So social prescribing isn’t just the sign posting, it’s the conversation, it’s the exploration of what’s going on for somebody . . . It is meaningful activity but again, not all social prescribers will understand meaningful activity . . . It’s to help people find occupational balance, but social prescribers won’t understand that. social prescribers jump is from ‘what I’ve heard’, to ‘what I think somebody needs’, but they’re not actually exploring the meaning of the activity to the person. (Participant 9)

I think that [occupational therapists] have that sort of fundamental, holistic understanding of occupational performance and the multiple dimensions of occupational performance . . . where we’re involved in social prescribing, it is far deeper and broader than just looking at social activities. We have the training to use activity analysis to consider the . . . complexity of the barriers. (Participant 6)

Finally, and to synthesise many of the points about a professional role which has more involvement in clinical assessment, management of complexity and with a ‘deeper and broader’ understanding of the link between activity and health, many participants highlighted a point of difference in relation to seniority and hierarchy:

when you’re working as a Band 7, you know, a lot of the signposting and I suppose the social prescribing doesn’t need to be done by somebody who’s a Band 7. (Participant 6)

## Making it work

The second of our over-arching themes explores how occupational therapists were working alongside social prescribing to manage workflow in their current roles, and how they imagined working in the future. Set against the background of challenges outlined in the first theme, the next theme is named *making it work* to reflect that occupational therapists were finding ways to make sense of both current practice and future ways of working.

### Managing workflow

Although there was heterogeneity in relation to team structures and models of service provision, there were also commonalities in the ways in which participants were managing workflow between occupational therapy and social prescribing.

Linked to the final extract in the theme above, occupational therapists discussed a hierarchical difference between the roles and it is therefore understandable that a strategy to manage workflow included delegation. Of particular interest in the extract below, Participant 7 refers to a traditional structure of occupational therapists and occupational therapy assistants to frame delegation of responsibilities:

And like I could see social prescribers sometimes being on the same kind of work as my OTA in terms of, if like social prescribers could run with something if they had the guidance. (Participant 7)

Furthermore, some participants reflected that this delegation to social prescribers would happen at a particular point in a pathway:

(I) try to use them as like a step down service. So we will do some intensive work with them to prepare them to work with social prescribing in the first place, and then we are like right, now we’ve done all this work, we will pass you on and step you down out into the community with the social prescribers. (Participant 14)

The term ‘step down’ was used by several participants and reflects earlier themes of occupational therapists proposing that they have a central role in managing complexity and that social prescribers may get involved during a subsequent and potentially more stable phase.

Many participants also discussed that the management of workflow should not be considered in terms of people needing one role or the other. Joint cases and joint visits were mentioned frequently:

I’ve had a few patients that I’ve gone out to see where they’ve got very . . . quite a lot of sort of, what I call clinical needs, which obviously I can address . . . so if we go out together and we can have a chat and we can identify what the needs are and then we obviously divide and do our own bits. (Participant 3)

Participants did voice challenges in relation to management of workflow. One area was the potential for duplication and the risk that this causes confusion for people accessing services:

Because there is that risk of us duplicating, trying to do the same thing with the person . . . because sometimes patients get referred here, there and everywhere and they’ve got loads of different people involved and it’s like who’s . . . Who are you? (Participant 5)

Another risk highlighted was the potential for social prescribers to be working outside of the boundaries of their role:

I think for me, probably the challenge is around exactly what I just said, around what’s clinical and what’s not clinical. I have to say, well, you’re now working in the realms of the clinical side of work, so perhaps one of us needs to do that on your behalf. (Participant 10)

Implied in the above extract is the importance of supervision and oversight, which was mentioned by many participants as an important strategy for managing workflow and delivering safe services, but an area where there was varying levels of involvement. In the first extract below, supervision is described as happening informally, although barriers to formal arrangements for supervision are highlighted in the second extract:

So, you know, if they’ve just come off the phone and it was a difficult case or they were concerned there was a safeguarding issue or they weren’t sure what the next step should be. So, we do a lot of that where they’re able to kind of get in touch with us and be like, oh this has just happened, you know, what’s your advice? Or what should I do next? Or who do I need to tell about this? (Participant 5)

No (not involved in supervision), because it is run by that different company. (Participant 14)

A further participant suggested a lack of clarity about arrangements and approaches to supervision for social prescribing workers:

What are their competencies, what do they need to be demonstrating, you know, what structure should that supervision have? (Participant 5)

### Imagining a positive future

Discussions about a positive future for occupational therapy and social prescribing often reflected examples of what was working well now, particularly in relation to co-working, joint cases and smooth workflow:

So, if I start a piece of work with somebody, I will do some social prescribing myself or if I think it is perhaps going to be a longer piece of work, I might co-work people or people may move between us in the team. So that’s, yeah, that’s how it’s working, how I envisage it (to) work in the future as well. (Participant 1)

Returning to the importance of personalised approaches and focussing on what matters most to people, many participants reflected on the natural allyship between occupational therapy and social prescribing based on shared values. They therefore imagined a future with occupational therapists advocating for this shared agenda:

I think as OTs we really understand what, how powerful that meaningful and purposeful activity can be. I think that’s the simplest way to put it and I think we, we’re in a position to advocate for that to support social prescribing. (Participant 2)

Involvement in supervision and leadership were revisited as part of reflecting on a positive imagined future, alongside involvement in recruitment, management and commissioning of services for local populations:

I think OTs could be really good at sort of managing and organising the service, thinking about identifying inclusion/exclusion criteria and barriers to participation and I think we are skilled at planning interventions and could be sort of well placed to be planning the community level interventions . . . it would be good to have a stronger presence within it. (Participant 17)

In general, participants suggested that better understanding of occupational therapy was an essential element of the profession working effectively and seamlessly alongside those in social prescribing roles. One participant voiced a call to action for professional bodies to strengthen the identity and position of the occupational therapy profession in relation to social prescribing, and then recognised the subsequent responsibility for organisations and individuals:

I’d like to see the Royal College of Occupational Therapists give a clear position statement that we do social prescribing. [They need] to put on training for staff. It then needs to come down to individual places of employment. So OT managers need to make sure their staff are clear on what their specific role is within that work remit . . . it needs to be in pre-registration standards, that we teach it as mandatory. (Participant 15)

## Discussion

This is the first study to our knowledge exploring the perceptions of occupational therapists and how they are contributing to the social prescribing agenda. Participants in this study were predominantly employed in primary care roles and were recruited to the study through self-identifying as being involved in social prescribing activities. Despite clear links to occupational therapy values and roles beyond primary care, this is an interesting finding to note and supports the reflection that social prescribing is gaining traction in primary care, potentially through the language and structures which are being created to support legitimacy and familiarity with the concept (Bradley and Scott, 2023).

Occupational therapists were able to share definitions of social prescribing, although subsequently positioning this definition in relation to their own profession was more problematic. Participants recognised that both roles started from foundations of what matters most to people, evidencing alignment with professional values to build from strengths-based, occupation-centred approaches (Whalley-Hammell, 2023) and policy drivers relating to personalised care (NHS, 2019). However, participants also articulated that they could not always fully enact this in their practice. Being unable to practice in a manner congruent with professional values has been linked to stress and burnout in occupational therapists (Walder et al., 2022), and emotional responses shared in this study may indicate such risks.

Occupational therapists created identities and positions in relation to social prescribing to potentially cope with challenges. Key positions emerged; positioning the profession as more senior, and positioning the profession as more medical. We have previously proposed that the theory of *Institutional Work* (Lawrence and Suddaby, 2006) provides an explanatory framework for how rules, boundaries and identities have aligned social prescribing with a medical model of health, and given authority to medical professionals (Bradley and Scott, 2023). Through this study, we note similarities in that occupational therapists are also trying to construct their own boundaries and create their own identities to legitimise authority and position.

To explore this positionality, it is important to acknowledge the social and political context influencing the creation of identity. For occupational therapists, this context is influenced by both historical challenges and contemporary developments. Well-documented challenges to occupational therapy identity from generic roles and ‘role creep’, where other professions assume some roles which are traditionally considered the domain of occupational therapy (Walder et al., 2022), were being discussed and reflected on in this study. There have also been examples where occupational therapists have felt the need to use language of dominant bio-medical models to develop identity and legitimise roles, potentially when their own language of occupation is not easily understood (Murray et al., 2015). With most participants in this study situated in primary care where a medical model of health is potentially shaping the social prescribing agenda (Bradley and Scott, 2023), alignment with a dominant discourse to be more ‘clinical’ than others in social prescribing roles is reflective of this.

Additionally, many participants were practising under the Additional Roles Reimbursement Scheme (ARRS) – a funding stream in the UK to support development of new roles in primary care (NHS England and NHS Improvement, 2019) – and struggles with identity, autonomy and contribution are reported elsewhere for this group (The Kings Fund, 2022). Professions named under this relatively recent ARRS scheme are facing unique opportunities from new funding and roles but with challenges of trying to evidence their value and make a distinct contribution in a competitive marketplace. Again, the desired positions of adding value by being ‘more clinical’ and providing value for money by being ‘more senior’ can further be understood through such reflection.

Delegation was an important mechanism supporting occupational therapists to manage workflow alongside social prescribing. Professional standards relating to delegation highlight the importance of the person who is being delegated to being competent in the identified activities (Royal College of Occupational Therapists [RCOT], 2021). Yet occupational therapists suggested a lack of clarity about competencies for social prescribing roles and suggested varying levels of oversight or involvement in supervision. The fact that a UK competency framework for social prescribing was only published in 2023 (after the data collection phase of this study) and includes limited specific reference to occupational therapy (NHS England, 2023) raises further questions about understanding of competence and safe delegation.

Employment and contracting arrangements were cited as challenging factors, particularly in relation to supervision. Approaches to contracting of ARRS roles in primary care have been compared to ‘supermarket sweep’, where those in leadership roles ran around and threw roles in the trolley because they were free (The Kings Fund, 2022; p. 9). It is important to acknowledge that Primary Care Networks were operating under uncertainty and pressure at this time, but such unplanned approaches were always likely to have consequences. For occupational therapists, this now includes confined positions in relation to supervision and influencing the growth of the social prescribing agenda. Interestingly, an overview of the international picture suggests some countries, rather than specifically developing new social prescribing roles, are adding the responsibilities to roles such as social workers and AHP (Morse et al., 2022). Yet explicit reference to occupational therapy in these examples is lacking and may once again reflect the profession having limited influence in wider developments.

Participants did reflect on their deep understanding of how people engage in occupations and their ability to look beyond one-size-fits-all reasoning processes to enable participation and mitigate against barriers and inequalities (Whiteford et al., 2021). For some, such reflections were already helping to create positive identities and positions, recognising that occupational therapy can benefit from social prescribing, and visualising involvement in developments as they move forwards. For others who were experiencing stress and negative emotions, such reflections are important reminders and can help with reframing and repositioning roles.

## Limitations

It was not the focus of this study to explore implementation of the occupational therapy role under the ARRS structure, yet this emerged as important and warrants further research. Similar studies which evaluate the integration of other professions in primary care systems have highlighted important opportunities, risks and recommendations (Eaton et al., 2021; Mills et al., 2022). Furthermore, despite sharing information to networks reaching all four home nations of the UK, all of our participants were based in England and Wales. Understanding the landscape across other nations of the UK, and internationally, is an area for further research. And whilst focussing on occupational therapists in the UK will not fully reflect the international picture, themes are likely to resonate with an international occupational therapy audience and can particularly support countries and services where developments are at different stages of maturity.

Our study focussed on the perceptions of occupational therapists about how they are contributing to the social prescribing agenda and how they perceive they could contribute to future developments. Exploring the perceptions of those in social prescribing roles, of other professionals and of people who access services would further extend understanding on this topic.

We suggest this exploratory study can be a foundation for research which evaluates effectiveness and acceptability of occupational therapy-led social prescribing interventions, and those offered by link workers. This would help inform commissioning of, and role-sharing within, services for local populations. Implementation and evaluation of professional development interventions for the occupational therapy and social prescribing workforce is another important focus for future practice development and research.

## Conclusion

Occupational therapists in this study perceived both alignment and points of distinction between their role and social prescribing although similarities and differences were often troublesome and contributing to questions about identity and legitimacy. Whilst recognising benefits in shared values of focussing on what matters most to people and connecting people to activities which promote health, there are risks within services such as duplication and lack of clarity about scope of practice. There are also risks to the profession if occupational therapy cannot articulate and evidence a unique contribution and value for money.

Practically, participants wanted clearer workflow and task-sharing between roles and saw supervision and professional development as integral to relationships and role development. Robust professional development activities and clear curriculum guidance are some of the practical ways professional bodies can support.

Together, occupational therapists and those in social prescribing roles have an opportunity to work collaboratively towards primary care and community services which address the social determinants of health. Opportunities for funding and wider role-creation are also likely to follow, and perhaps more importantly, services orientated to outcomes that are meaningful to people who access services.

**Key findings**

- Occupational therapists see alignment and difference between their own profession and social prescribing values and roles.
- One challenge of alignment is questions about identity and legitimacy for occupational therapists.
- Some occupational therapists are now involved in supervision, recruitment and professional development of social prescribers but are asking for clearer guidance and structures to support them in these roles.

**What the study has added**

This study has developed understanding about how developments in social prescribing in the UK have been experienced by occupational therapists and how occupational therapists view their own contribution to this agenda moving forwards.

## Supplemental Material

sj-docx-1-bjo-10.1177_03080226241270520 – Supplemental material for Occupational therapist’s involvement in social prescribing: A qualitative interview study Supplemental material, sj-docx-1-bjo-10.1177_03080226241270520 for Occupational therapist’s involvement in social prescribing: A qualitative interview study by Gemma Bradley, Beth Atkin, Helen Atkin and Jason Scott in British Journal of Occupational Therapy
