# Supplementary material for: Occupational therapist’s involvement in social prescribing: A qualitative interview study
Source: Br J Occup Ther. 2024 Aug 19;88(1):25–34. doi: 10.1177/03080226241270520 (PMC11887915; doi:10.1177/03080226241270520)
Supplement: sj-docx-1-bjo-10.1177_03080226241270520 – Supplemental material for Occupational therapist’s involvement in social prescribing: A qualitative interview study [file sj-docx-1-bjo-10.1177_03080226241270520.docx]

**Researcher Introduction**

- Introductions – researcher from ***
- Thanks for participation
- Revisit the aims
  - - To explore how occupational therapists are currently contributing to social prescribing developments
    - To explore how occupational therapists perceive they could contribute to furthering the social prescribing agenda
- If there are any questions you would prefer not to answer, just let me know and we can move on.
- It will last between 30-60 minutes, depending on how much you have to say. If at any point you want to take a break just let me know.
- Their rights as participants
  - - Right to withdraw at any time
    - Ask questions at any time
    - Right to complain
    - What you tell me today will remain completely confidential and we will redact identifiable information. However if you tell me something that suggests yourself or someone else may be at risk of harm, I will have to tell someone
  - Remind them that the interview will be recorded
  - Have they got any questions before we start?

1. **YOUR ROLE**

I would like to start with a few questions about your role…

- 1. What is your job title?
  2. Which geographical area do you work in? (perhaps give options organised by ICS*)
  3. What typical patient groups do you work with?
     1. How does a typical patient present?
     2. Can you tell me about the needs of a typical patient?
  4. What patient groups/populations do you work with?
  5. How long have you been practicing as an occupational therapist?
  6. Do you have a grade (or how would you compare your grade to an NHS band?)? (NB this may already be known from participant recruitment – don’t need to ask again)
  7. Are you employed by…NHS…Social care…directly by a GP practice…Private…VSCE?
  8. When your role was advertised – what previous experience/educational level/professional qualifications were required (if you can remember)?
  9. Can you say a little bit about the structure of the service you work within – e.g. who you are managed by/whether you manage others/how many other people do the same role?
  10. And can you say a little bit about why you came in this role?

1. **YOUR ROLE IN RELATION TO SOCIAL PRESCRIBING**
   1. Social prescribing can mean many things to different people – how do you describe it?
   2. How would you describe your role in relation to social prescribing?
      1. How did you get involved in this role – did you apply for a new role or was it part of an existing role?
   3. Have you undertaken any extra development activities specifically related to social prescribing?
   4. Do you work alongside others who you would identify to have social prescribing roles? If YES – can you tell us about…
      1. Their job title – is it the same as yours?
      2. Their employing organisation – different or the same as yours?
      3. Their grade (or NHS grade equivalent)?
      4. Can you tell me a little more about how you work together?
         1. What are the similarities and differences between your roles?
      5. How do you think occupational therapy is perceived within your team?
2. **DESCRIBING AND DIFFERENTIATING ROLES**

We have slightly different questions depending on whether you primarily see yourself as an occupational therapist who works alongside others in social prescribing roles, or whether you are responsible for social prescribing yourself. Which of these roles do you primarily see yourself as? Or do you see yourself as doing both? If you do both – how do you balance both of these elements within your role?

**Either ask SET 3a or SET 3b questions**

3a. If you see your main role as an **occupational therapist** who works alongside others in social prescribing roles…

- 1. How do you feel your occupational therapy skills and knowledge support you in your social prescribing related role?
  2. How would you describe your involvement with other people who are in social prescribing roles – can you give any examples of any of the activities below?
     - Recruitment of people to social prescribing roles
     - Education and development of people in social prescribing roles
     - Working on joint cases with people in social prescribing roles
     - Supervision, mentoring or coaching of people in social prescribing roles
     - Line management
     - Writing business cases/commissioning
     - Service design (e.g. looking at gaps in service provision)

3b. If you see your main role as an **occupational therapist delivering social prescribing yourself…**

- 1. How do you feel your occupational therapy skills and knowledge support you in your social prescribing related role?
  2. How do your occupational therapy values link to this role?
  3. Can you describe aspects of professional education and training which are helping you in this role?

1. **MODELS OF SERVICE DELIVERY**
   1. Can you describe your experience of pathways between occupational therapy and social prescribing?
   2. In your experience, what is the key difference between occupational therapy and social prescribing?
   3. Can you give examples of where the model of service provision involving occupational therapy and social prescribing is working well?
   4. What are the main challenges with models of service provision involving occupational therapy and social prescribing? Can you give examples of challenges? What would help to overcome these challenges?
      1. Do any of the challenges relate to the supply of activities? How do you influence this as an occupational therapist?
   5. How do you use your occupational therapy skills to navigate challenges?
   6. What would you like to see in terms of occupational therapy involvement in social prescribing in the future? How would you like to see this working?

Is there anything else that we haven’t asked you that you would like to tell us about?

Do you know of any other individuals or networks who might be interested in being involved in the project? Could you forward information on?
